# Why do experts on ectodermal dysplasia (ED) meet again?

**DOI:** 10.1186/1746-160X-8-S1-I1

**Published:** 2012-05-25

**Authors:** Birgitta Bergendal

**Affiliations:** 1 National Oral Disability Centre for Rare Disorders, Jönköping, Sweden

## 

Hypohidrotic ectodermal dysplasia was first described in the mid 19^th^ century by John Thurnam. In 1875, Charles Darwin described the signs and symptoms in a four-generation family, “the ten men from Scinde”, explaining X-linked inheritance at the same time when Gregor Mendel mapped the laws of inheritance. The typical clinical expression in boys with X-linked hypohidrotic ED makes this syndrome easy to recognize, although usually not in the newborn, where the vulnerability for overheating is at its peak. Still a majority of these boys seems to be diagnosed by dentists, either when no teeth erupt on time or when the first teeth show up in an unusual position and with an aberrant form. In many of the other >190 different ED forms, there are even greater difficulties in getting to a diagnosis.

In recent years, the internet has had an enormous impact on access to information. The successful initiative of Mary Kaye Richter, who thirty years ago started the first support group in the US, has had many followers, and today the International ED network (IEDN) covers four continents. However, individuals and families with ED suffer the same difficulties that are typical for all rare disorders in search for physicians and dentists who can help, treat and give advice for good quality of treatment to assure a good quality of life. Recent research has led to a better understanding of symptoms not only in ectodermally derived tissues, but also in tissues of mesenchymal origin. The last decade has seen a veritable eruption of new knowledge, not only in the genetic field, but also in diagnostics and treatment. The most spectacular scientific contribution was when X-linked hypohidrotic ED as the first heritable disease was shown to be permanently corrected by treatment with the missing protein. Now we are following the application of these findings from mice to men. This also calls for estimations of prevalence, ethical considerations, more research and hope for a better future for individuals with ED.

